# Early life stress and macaque amygdala hypertrophy: preliminary evidence for a role for the serotonin transporter gene

**DOI:** 10.3389/fnbeh.2014.00342

**Published:** 2014-10-06

**Authors:** Jeremy D. Coplan, Hassan M. Fathy, Andrea P. Jackowski, Cheuk Y. Tang, Tarique D. Perera, Sanjay J. Mathew, Jose Martinez, Chadi G. Abdallah, Andrew J. Dwork, Gustavo Pantol, David Carpenter, Jack M. Gorman, Charles B. Nemeroff, Michael J. Owens, Arie Kaffman, Joan Kaufman

**Affiliations:** ^1^Department of Psychiatry and Behavioral Sciences, State University of New York, Downstate Medical CenterBrooklyn, NY, USA; ^2^Departamento de Psiquiatria, Neuroradiology, Universidade Federal de São PauloSão Paolo, Brazil; ^3^Departments of Psychiatry, Neuroscience, and Radiology, Mount Sinai School of MedicineNew York, NY, USA; ^4^Psychiatry, New York State Psychiatric InstituteNew York, NY, USA; ^5^Mental Health Care Line, Michael E. Debakey VA Medical CenterHouston, TX, USA; ^6^Menninger Department of Psychiatry and Behavioral Sciences, Baylor College of MedicineHouston, TX, USA; ^7^Department of Psychiatry, Mount Sinai School of MedicineNew York, NY, USA; ^8^Department of Psychiatry, Yale University School of MedicineNew Haven, CT, USA; ^9^Clinical Neuroscience Division, National Center for PTSDWest Haven, CT, USA; ^10^Department of Molecular Imaging and Neuropathology, New York State Psychiatric InstituteNew York, NY, USA; ^11^Departmets of Psychiatry and Pathology and Cell Biology, College of Physicians and Surgeons of Columbia UniversityNew York, NY, USA; ^12^Comprehensive NeuroScience CorporationWestchester, NY, USA; ^13^Department of Psychiatry and Behavioral Sciences, University of Miami Health SytemsMiami, FL, USA; ^14^Department of Psychiatry and Behavioral Sciences, Emory University School of MedicineEmory, GA, USA; ^15^Department of Psychiatry, Yale University School of MedicineNew Haven, CT, USA

**Keywords:** amygdala, early life stress, non-human primates, MRI, stress, serotonin transporter gene

## Abstract

**Background:** Children exposed to early life stress (ELS) exhibit enlarged amygdala volume in comparison to controls. The primary goal of this study was to examine amygdala volumes in bonnet macaques subjected to maternal variable foraging demand (VFD) rearing, a well-established model of ELS. Preliminary analyses examined the interaction of ELS and the serotonin transporter gene on amygdala volume. Secondary analyses were conducted to examine the association between amygdala volume and other stress-related variables previously found to distinguish VFD and non-VFD reared animals.

**Methods:** Twelve VFD-reared and nine normally reared monkeys completed MRI scans on a 3T system (mean age = 5.2 years).

**Results:** Left amygdala volume was larger in VFD vs. control macaques. Larger amygdala volume was associated with: “high” cerebrospinal fluid concentrations of corticotropin releasing-factor (CRF) determined when the animals were in adolescence (mean age = 2.7 years); reduced fractional anisotropy (FA) of the anterior limb of the internal capsule (ALIC) during young adulthood (mean age = 5.2 years) and timid anxiety-like responses to an intruder during full adulthood (mean age = 8.4 years). Right amygdala volume varied inversely with left hippocampal neurogenesis assessed in late adulthood (mean age = 8.7 years). Exploratory analyses also showed a gene-by-environment effect, with VFD-reared macaques with a single short allele of the serotonin transporter gene exhibiting larger amygdala volume compared to VFD-reared subjects with only the long allele and normally reared controls.

**Conclusion:** These data suggest that the left amygdala exhibits hypertrophy after ELS, particularly in association with the serotonin transporter gene, and that amygdala volume variation occurs in concert with other key stress-related behavioral and neurobiological parameters observed across the lifecycle. Future research is required to understand the mechanisms underlying these diverse and persistent changes associated with ELS and amygdala volume.

## Introduction

Early adversity, including experiences of institutional care, abuse and neglect, and parenting compromised by psychiatric illness, is a major risk factor for the development of anxiety and mood disorders later in life (Warner et al., 2008; Slopen et al., 2012; Lindert et al., 2014). Several observations suggest that early life stress (ELS) increases vulnerability to adult psychopathology by altering amygdala function (Tottenham and Sheridan, 2009). The amygdala plays a key role in regulating fearful response and undergoes major developmental changes during the early postnatal period (Payne et al., 2010). Compared to non-institutionalized controls, Romanian adoptees with severe early institutional deprivation have been reported to have enlarged amygdala volumes (Mehta et al., 2009). In another cohort of institutional reared children, enlarged amygdala volume was restricted to children adopted when they were older than 15 months old. Children who were placed in institutional care and were adopted earlier in life did not differ from controls (Tottenham et al., 2010), suggesting a potential period of plasticity and recovery in early development. Enlarged amygdala volume has also been reported in maltreated adolescents, (Whittle et al., 2013) and children of mothers presenting with consistent depressive symptomatology over the course of the child's life (Lupien et al., 2011). In this latter study (Lupien et al., 2011), a significant positive correlation was observed between mothers' mean depressive scores and the children' amygdala volumes, underscoring the importance of maternal-infant attachment disruption and compromised parenting in the pathogenesis of amygdala enlargement.

In response to chronic stress, rodents exhibit enhanced dendritic arborization in the basolateral complex (BLA) of the amygdala in addition to exaggerated amygdala activity (Vyas et al., 2002, 2004). The latter neuronal modification in rodents exposed to chronic stress was associated with an increase in anxiety-related behavior and enhancement of fear conditioning (Conrad et al., 1999; Sandi et al., 2003; Vyas et al., 2004; Wood et al., 2008; Roozendaal et al., 2009). Rhesus monkeys with bilateral lesions of the central nucleus of the amygdala (CeA), the source of the amygdala's fear-related efferent pathways, displayed significantly less fear-related behavior as well as decreased concentrations of CSF corticotropin releasing-factor (CRF) in comparison to controls (Kalin et al., 2004), further highlighting the role of the amygdala in mediating anxiety behaviors and modulation of stress reactivity. Recent rodent studies have demonstrated that stress-induced modifications of the hypothalamic–pituitary–adrenal axis shape amygdala–mPFC circuitry and ELS accelerates precocious maturation of these circuits (Gee et al., 2013).

Exposure to ELS in humans can be variable and entails a complex interaction among multiple factors (Kaufman et al., 2004). Using animal models, we can experimentally control the characteristics of ELS, provide objective measures of environmental adversity, and prevent exposure to confounding levels of antenatal stress (Malter Cohen et al., 2013) and stress following ELS (Rosenblum and Paully, 1984). Moreover, the shorter life span of macaques compared to humans facilitates longitudinal assessment, thus allowing the examination of the effects of ELS into adulthood (Jackowski et al., 2011). One paradigm of early-life stress exposure in the bonnet macaque can be achieved through imposing unpredictable foraging conditions on the mother—a procedure termed variable foraging demand (VFD) in contrast to normative low foraging demand (LFD)—thereby disrupting normative patterns of maternal contingent responsivity and infant attachment (Rosenblum and Paully, 1984; Jackowski et al., 2011).

The primary goal of this study was to examine amygdala volume in VFD-reared and normally reared bonnet macaques. To the best of our knowledge, the effect of ELS on amygdala size has not been reported yet for nonhuman primates. Because there is preliminary evidence that certain of these biological alterations are moderated by genetic variation in the serotonin transporter gene (5-HTTLPR) polymorphism (Barr et al., 2003, 2004; Alexander et al., 2012; Spinelli et al., 2012), the effect of 5-HTTLPR genotype on amygdala volume was also explored. Secondary analyses were then conducted examining the association between amygdala volume and other biological and behavioral alterations associated with ELS. In prior studies by our group and others, ELS has been linked to multiple biological abnormalities associated with stress-related psychiatric disorders, including: increased CSF concentrations of the stress hormone CRF (Coplan et al., 1996); reduced fractional anisotropy (FA) in the anterior limb of the internal capsule (ALIC) (Coplan et al., 2010)—an important white matter tract for thalamo-frontal connections (Hazlett et al., 2012), and site of deep brain stimulation for treatment resistant depression (Gutman et al., 2009)—and reduced hippocampal neurogenesis (Perera et al., 2011), a marker sensitive to stress and the neurotrophic effects of antidepressant medications (Duman and Monteggia, 2006). Excess rates of timidity responses to a human intruder in VFD-reared subjects possessing the short allele have been reported (Jackowski et al., 2011).

## Materials and methods

### Subjects

Imaging data were available for 21 adult male Bonnet Macaques *(Macaca radiata*): 12 VFD-reared and 9 normally reared subjects. At time of MRI scanning, there were no statistical differences between the groups in age (VFD: 60.04 ± 32.27 months; non-VFD: 66.23 ± 31.7; *t* = 0.44; *df* = 19; *p* = 0.66) or weight (VFD: 4.87 ± 1.31 kg; non-VFD: 5.15 ± 1.78, *t* = 0.42, *df* = 19; *p* = 0.68).

### Procedures

**Figure 1:**
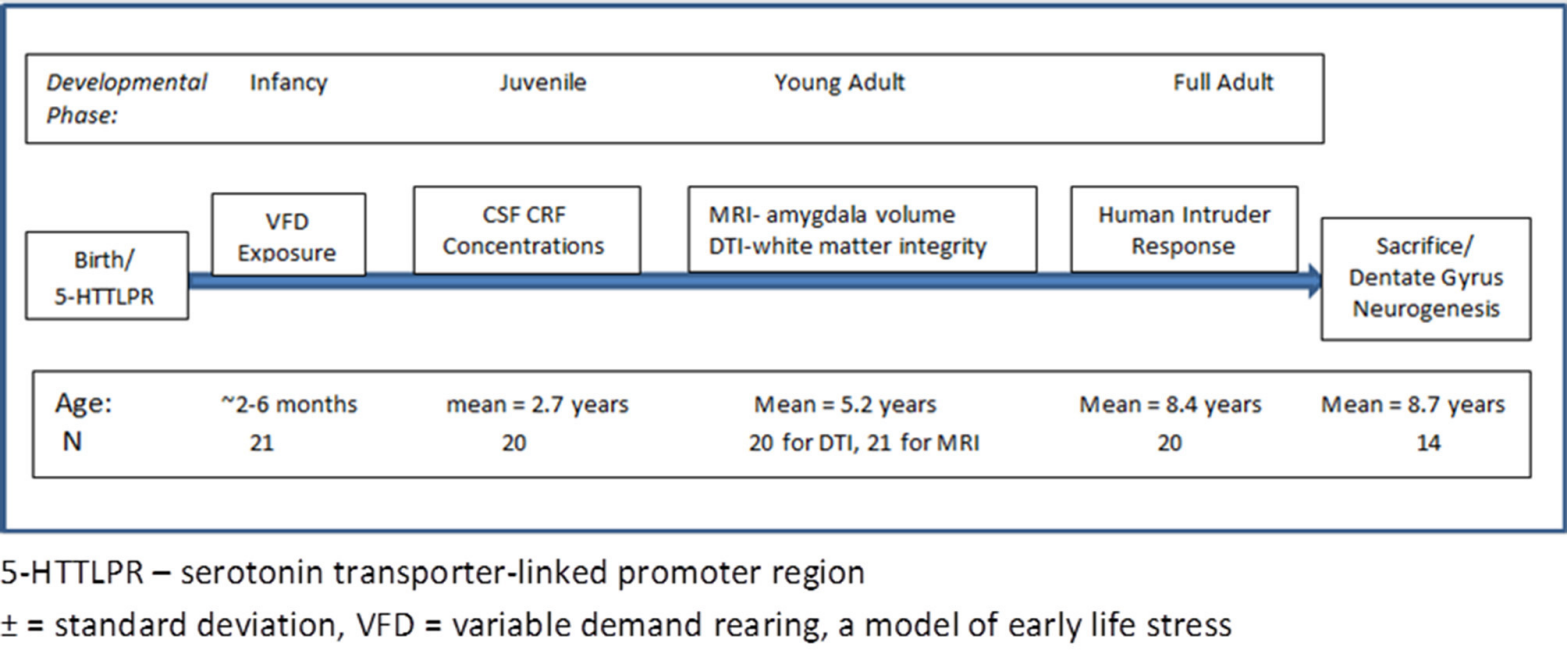
**Time line and experimental procedure of the study**. The figure outlines the time line of the developmental phase of the bonnet macaque male subjects and at which point interventions were performed. The bottom row provides the age of the subjects at the time of the experimental intervention.

A time line for all procedures the animals in this cohort experienced is provided in Figure 1. For the imaging studies, nonhuman primates were socially-housed in the SUNY-Downstate Nonhuman Primate Facility. The study was approved by the Institutional Animal Care and Use Committees of SUNY-Downstate, Columbia University/New York State Psychiatric Institute, Mount Sinai Medical School, and Veterans Administration West Haven Connecticut campus.

### Rearing procedures

Mother-infant dyads were group-housed in pens of 5–7 dyads each, and stabilized for at least 4 weeks prior to VFD onset (Coplan et al., 1996). After infants reached at least 2 months of age, dyads were subjected to a standard VFD procedure that involved 8 alternating 2-week blocks in which maternal food was readily obtained (LFD) or more difficult to access (high foraging demand; HFD). During HFD conditions, the mothers had to find food by digging through clean wood-chip in a foraging cart. Food can be accessed by mothers through apertures in the sides of the foraging cart. In the control non-VFD condition, the mothers' food access was continuous. Adequate food was always available under both conditions, and there were no differences in weight between VFD and non-VFD mothers or infants. However, the unpredictability of foraging conditions putatively prevented VFD mothers from adequately attending to their infants. The early-life stress paradigm putatively occurs through the disruption of normative patterns of maternal rearing and infant attachment (Coplan et al., 2005). After infancy, no experimental manipulations occur that may confound the VFD-rearing effects.

### Scanning procedure

As described previously (Jackowski et al., 2011), on the day of the brain scan, study subjects were ushered into familiar carrying cages and transported to Mount Sinai Medical Center. Subjects were transported in a dedicated animal transport van with air-conditioning. Upon arrival at the scanner, animals were transported to a squeeze cage and following a brief restraint period, were rapidly given anesthetic agent intramuscularly. Saffan®, previously known as CT1341, is an injectable steroid anesthetic for use in cats and monkeys and because it minimizes motion artifact as compared to ketamine, the drug was used to conduct the scans. Saffan® was administered at a dose of 16 mg/kg, which comprises two bioactive constituents; 12 mg/kg of alphaxalone and 4 mg/kg alphadolone acetate. Infrequently, if there was evidence of motion during the scan secondary to diminished level of anesthesia, animals necessitated subsequent doses of Saffan® (¼ initial dose). Once sedated the monkeys' heads were positioned in a Styrofoam headrest inside a human knee coil taped snugly over the forehead to minimize movement. Subjects remained anesthetized throughout scanning and were continuously monitored by pulse oximeter. Subjects usually awakened within 20 min following completion of the 1 h scan. Following the imaging procedures, subjects returned on the same day to their home cages.

The MRI data were acquired in a 4-T Siemens Allegra head-only scanner. The protocol for the structural scans consisted of a three-plane sagittal localizer from which all other structural scans were prescribed. The following structural scans were acquired: axial 3D-MPRAGE (*TR* = 2500 ms, *TE* = 4.4 ms, *FOV* = 21 cm, matrix size = 256 × 256, 208 slices with thickness = 0.82 mm); Turbo spin echo T2-weighted Axial (*TR* = 5380 ms, *TE* = 99 ms, *FOV* = 183 × 21 cm, matrix = 512 × 448, Turbo factor = 11,28 slices. thickness = 3 mm, skip 1 mm).

### MRI data pre-processing and analysis

**Figure 2:**
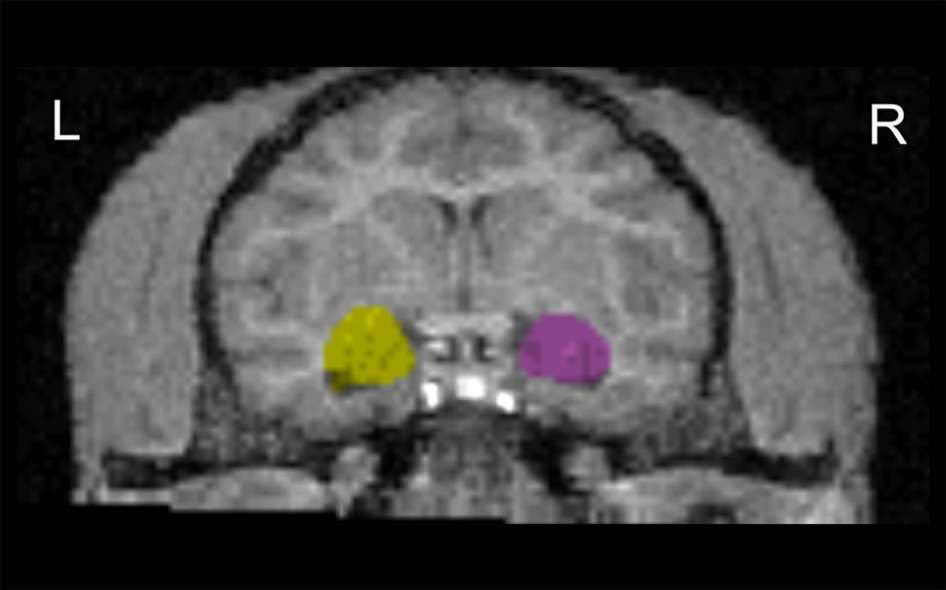
**Coronal view of the amygdala outline in T1-weighted images**. Images were aligned to the hippocampus axis. L, left hemisphere; R, right hemisphere.

As described previously (Jackowski et al., 2011) (Figure 2), all MRI regions of interest (ROI) analyses were completed by raters blind to subjects' rearing and genotype. The axial MPRAGE series were imported into ANALYZE AVW 7.0 (Biomedical Imaging Resource, Rochester, MN) (Robb et al., 1989) software platform and converted to cubic voxel dimensions of 0.44 mm using a cubic spline interpolation algorithm. The amygdalae were manually traced using a detailed set of guidelines developed by Schumann et al. (2004) and adjusted, when necessary, to the bonnet macaque brain morphology using a primate brain atlas (Wu and Robertson, 2000). The tracings were performed in oblique coronal slices, but were also checked in sagittal and axial views. Repeated measurements were performed in a random order on 5 subjects, and both intra-rater and inter-rater reliability gave an ICC > 0.91 for volumetric assessments. In order to isolate whole brain from its surroundings, skull, surface CSF, and meninges were stripped using a level-set gray-white algorithm which includes a combination of tools as image thresholding, region growing, and manual tracing (for details see Zeng et al., 1999).

### 5-HTTTLPR genotyping

Genotypes were determined by PCR amplification followed by size fractionation on a 2% agarose gel (Jackowski et al., 2011). Primers used were CAG CAC CTA ACC CCC TAA TGT CCC TG and GAT TCT GGT GCC ACC TAG ACG CCA G. Each 10 μl reaction contained 20 ng DNA, 1 μM of each primer, 1 M betaine, 10 μM dNTPs, and 0.1 unit KlenTaq polymerase, in manufacturer's PC2 buffer. Cycling parameters were 95°C for 5 min followed by 30 cycles at 95°/72° for 30 and 60 s respectively, using an MJR thermal cycler.

### CSF sampling

Previously reported data on CSF CRF concentrations obtained when the primates were adolescents and a mean of 2.7 years old (Coplan et al., 2011) were available on seven LFD and 11 VFD. The time between the end of the VFD procedure and the CSF sampling was about 2–3 years. Subjects were taken from their home cages and placed in carrying cages, a routine procedure. For CSF sampling, subjects were released into restraint cages and intramuscular ketamine (15 mg/kg) was administered. CSF samples were then placed in Gant tubes and stored in a −70°C freezer. Assays for CRF were performed according to the methods described in Nemeroff et al. (1984). The assay has a sensitivity of 2.5 pg per tube and intra- and inter-assay coefficients of variation of 3–6% and 10–13%, respectively. The laboratory personnel conducting the CRF radioimmunoassays were blind to the subjects' rearing status.

### Diffusion tensor imaging

DTI data were acquired on the 3-T MRI Siemens Scanner (Coplan et al., 2010) using a pulsed-gradient spin-echo sequence with EPI-acquisition on the same day the structural data were obtained (*TR* = 4100 ms, *TE* = 80 ms, *FOV* = 21 cm, matrix = 128 × 128, 24 slices, thickness = 3 mm skip 1 mm, b-factor = 1250 s/mm2, 12 gradient directions, 5 averages). Raw DTI data were transferred to an off-line workstation for post-processing. In-house software written in Matlab v6.5 (The Mathworks Inc. Natick, MA) was used to compute the anisotropy and vector maps. The FA images were then converted to analyze format. MEDx v3.4.3 software (Medical Numerics Inc., Sterling, VA) was used to inspect and define ROIs on the FA images. Primary ROI included the anterior limb of the left and right internal capsule.

### Behavioral measures of emotional reactivity

Twenty of the 21 animals that participated in this MRI study were subject to behavioral testing during late adulthood, approximately 3 years after neuroimaging. Each animal was individually exposed briefly to an intruder, a fear-stimulus which is a variation of a previously detailed masked intruder paradigm (Rosenblum et al., 2001). Males were singly housed in holding cages and the “intruder” entered into the pen and stood about six feet in front of the cage making direct eye contact with the monkey. Emotional responsivity was rated by two experimenters blind to amygdala volume and rearing status using a 3-point scoring scale. To receive a score of one for intruder distress, subjects exhibited “confrontational” behaviors including; fang-baring, growling, direct eye contact, pilo-erection, ear flexing, cage shaking, and mouth gaping. The least distressed response received a score of “three” which was characterized by an animal that was minimally confrontational, averting eye contact, submissive and displaying lip-smacking, receding to the back of the cage and exhibiting “timidity” in response to the intruder. A score of two describes a subject with intermediate or alternating levels of both confrontational and timid behaviors. One hundred percent inter-rater reliability was observed for the intruder behavioral scoring system.

### Neurohistochemistry

Using previous methods (Perera et al., 2007) the subjects were anesthetized to a surgical depth with sodium pentothal and transcardially perfused with saline. The brains were removed and post-fixed in 4% paraformaldehyde for immunnohistochemical staining and analysis. The left hippocampus was cut into 40 μm sections and every 40th section was immunostained to detect cells labeled with the immature neuronal marker doublecortin (DCX) using a mouse the monoclonal primary antibody (1:3000; Santa Cruz). The secondary antibody was biotinylated horse anti-mouse IgG (1:200; Vector Laboratories) visualized with avidin-biotin complex solution (Vector) and diaminobenzidine (DAB; Sigma,). The density of DCX-labeled cells per mm^3^ of the subgranular zone (SGZ) was estimated for each animal. Two independent raters, masked to treatment condition counted all of unambiguously DCX-labeled cells in the SGZ of the dentate gyrus (defined as a two-cell-body-wide zone on either side of the border of the granule cell layer) using a 40× objective.

### Statistical analysis

Amygdala volumes were shown to be normally distributed using the Kolmogorov-Smirnov test. One outlier, a non-VFD subject exhibited value 3.44 SD above the mean left amygdala volume and was excluded from subsequent analyses. Brain volume predicted overall amygdala volume [*F*_(1, 15)_ = 17.32; *p* = 0.0008] and was therefore used as a covariate for all analyses entailing amygdala volume. Because all subjects were male, controlling for sex effects was not necessary; age and weight were also not used as covariates for the primary analyses as they were not associated with amygdala volume. For determination of rearing effects for amygdala volume, a general linear model (GLM) was conducted using left and right amygdala volume as the repeated measure dependent variables. We used rearing group (VFD vs. non-VFD) as a categorical variable and brain volume as a continuous independent variable or covariate. The GLM was followed by univariate analyses for side effects when significant effects were not evident on the overall GLM for overall amygdala volume. To determine whether an effect of the serotonin transporter gene was evident, we repeated the GLM but included serotonin transporter gene polymorphism (long “ll” vs. short allele “sl”) as a categorical factor. Their factorial interaction [gene^*^environment (G × E) effect] was determined and *post-hoc* Fisher least-square difference testing was performed should the G × E interaction prove to be significant.

Neither age nor weight predicted the secondary measures (timidity, CSF CRF concentrations, anterior limb of internal capsule FA, and hippocampal neurogenesis) and were therefore not used as covariates. For behavioral measures, we used a GLM with behavioral response scores (Warner et al., 2008; Slopen et al., 2012; Lindert et al., 2014) as the categorical variable and amygdala volume as the repeated measures dependent variable with brain volume as a covariate followed by univariate analyses for side effects. For CSF CRF concentrations, rearing groups were merged and using a median split, subjects were divided into “high” vs. “low” CRF groupings as the categorical variable in the GLM followed by univariate analyses for side effects. For white matter effects, we merged both rearing groups and conducted a GLM using mean FA of the left and right ALIC as the continuous independent variable with brain volume as a covariate and right and left amygdala as dependent repeated measures. *Post-hoc* Pearson's correlations were performed to ascertain the relationship between FA and amygdala volume. Finally, using a GLM we examined the relationship of neurogenesis (as reflected by dentate gyrus doublecortin staining) as a continuous independent variable to right vs. left amygdala volume as the repeated measures dependent variable and brain volume as a covariate followed by univariate analyses. Pearson's correlations were performed to examine the relationship between neurogenesis and amygdala volume. For each of the secondary measures in which new groups were formed, the frequency of subjects with either VFD-rearing or VFD rearing accompanied by the short allele of the serotonin transporter gene was compared in the “stress-related” vs. “non-stress related” groups. A Friedman ANOVA and Kendall Coefficient of Concordance for multiple dependent variables was performed to test the hypothesis of an overall “master” effect affecting multiple diverse biobehavioral parameters following ELS. All tests were two-tailed with a significance probability designated as *p* = 0.05. Correction for multiple testing was not performed because of the exploratory nature of the study.

## Results

### Primary analyses

#### Amygdala volume

Although there was only a trend for a group effect [*F*_(1, 17)_ = 3.77, *p* = 0.068] on the repeated measures GLM, univariate analyses revealed a significant group effect on the left side for amygdala volume [VFD mean = 0.37 cm^3^; 95% *CI*: 0.34–0.39; *N* = 12 vs. non-VFD mean = 0.31 cm^3^; 95% *CI*: 0.28–0.34; *N* = 8; *F*_(1, 17)_ = 5.51; *p* = 0.031, partial eta-squared = 0.24]. Results on the right side were non-significant [*F*_(1, 17)_ = 1.16; *p* = 0.24].

#### Serotonin transporter gene effects

**Figure 3:**
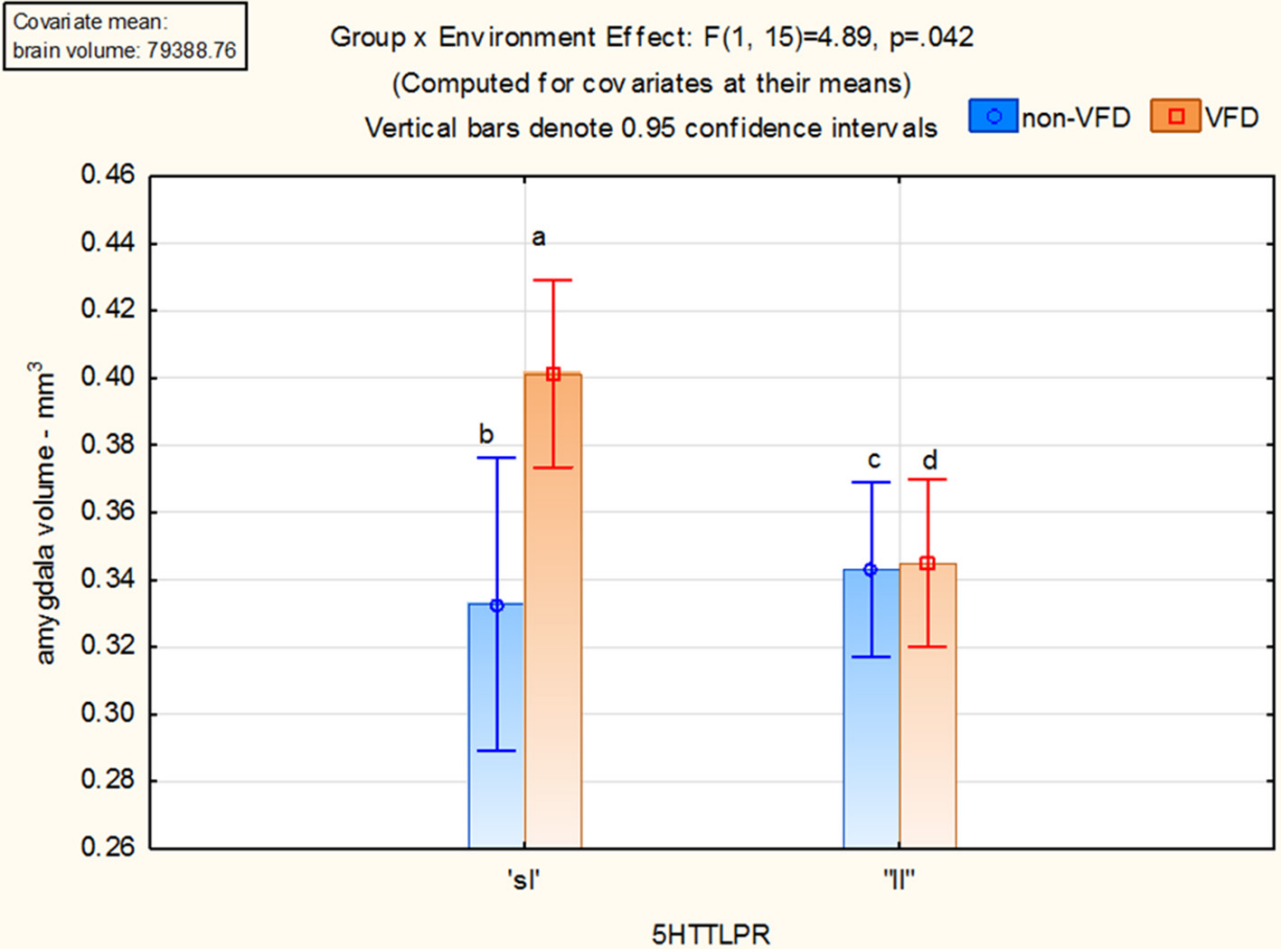
**Gene × environment effect for amygdala volume in adversely-reared macaques possessing the short allele of the serotonin transporter gene**. Using the GLM factorial design, a group effect for overall amygdala volume was noted [*F*_(1, 15)_ = 5.65; *p* = 0.031] but no significant allele effect. A gene by environment effect [*F*_(1, 15)_ = 4.89; *p* = 0.042] was noted. Using Fisher least-square differences *post-hoc* testing, VFD-reared macaques expressing the short allele of the serotonin transporter gene exhibited larger amygdala volume compared to unstressed controls with the “sl” allele [a > b; *p* = 0.019] or “ll” [a > c; *p* = 0.002] allele subjects and at a trend for ELS subjects with the long allele [a > d; *p* = 0.088]. Two subjects were non-VFD “sl” whereas six subjects were non-VFD “ll.” Five subjects were VFD “sl” and seven subjects were VFD “ll.”

With the one outlier removed, 20 subjects were available for analysis. No subjects possessed the “ss” allele. Two subjects were non-VFD “sl” whereas six subjects were non-VFD “ll.” Five subjects were VFD “sl” and seven subjects were VFD “ll.” Using the GLM factorial design, a group effect for overall amygdala volume was noted [*F*_(1, 15)_ = 5.65; *p* = 0.031] but no significant allele effect was evident. As depicted in Figure 3, a gene by environment effect [*F*_(1, 15)_ = 4.89; *p* = 0.042] was noted. Using Fisher least-square differences *post-hoc* testing, VFD-reared macaques expressing the short allele of the serotonin transporter gene exhibited larger amygdala volume compared to unstressed controls with the “sl” [*p* = 0.019] or “ll” [*p* = 0.002] genotype allele subjects and at a trend level for VFD subjects with the long or “ll” allele [*p* = 0.088] (see Figure 3). A gene × environment effect was not observed for right or left amygdala when analyzed separately.

### Secondary analyses

#### Behavioral response to an intruder

Three subjects exhibited confrontational behaviors, eight, intermediate behaviors, and eight, timid behaviors. Since mean amygdala volumes were not different between the confrontational and intermediate group, those groups were collapsed into a “non-timid” group. Using GLM, there was an overall group effect, with the subjects who exhibited timid behaviors displaying larger amygdala volumes than subjects who exhibited confrontational and intermediate responses [*F*_(1, 15)_ = 6.68; *p* = 0.019; timid subjects mean = 0.38 cm^3^; 95% *CI*: 0.35–0.40; *N* = 8 vs. non-timid subjects mean = 0.34 cm^3^; 95% *CI*: 0.32–0.36; *N* = 11]. Although there were no differences between VFD representation in the timid vs. non-timid group, four of the timid group were “s” allele subjects exposed to VFD whereas only one of 11 were VFD “s” subjects in the non-timid group [χ^2^ = 4.00; *df* = 1, *p* = 0.045].

#### CSF CRF concentration

Neither age nor weight was related to CSF CRF concentrations in this sample (Jackowski et al., 2011). Eight subjects were included in the “high” CRF group whereas nine subjects comprised the “low” CRF group. The mean CSF CRF concentration in the high group was 149.17 ± 64.68 ng/ml vs. 84.52 ± 12.30 ng/ml in the low group [*t* = 2.95; *df* = 15; *p* = 0.0099]. The “high” CRF group exhibited enlarged amygdala [mean = 0.37 cm^3^; 95% *CI*: 0.35–0.40 (*N* = 8)] in comparison to the “low” CRF group [mean = 0.33 cm^3^; 95% *CI*: 0.30–0.36 (*N* = 9): *F*_(1, 14)_ = 4.66; *p* = 0.048]. Using continuous measures, and controlling for brain volume, CSF CRF concentrations were positively associated with amygdala volume [*F*_(1, 14)_ = 8.24; *p* = 0.012]. This effect was significant for right [*F*_(1, 14)_ = 6.32; *p* = 0.025] and left amygdala[*F*_(1, 14)_ = 6.75; *p* = 0.021]. The composition of the “high” vs. “low” CRF group did not differ with respect to VFD frequency. However, four of the eight “high” CRF group were VFD who possessed the “s” allele of the 5-HTTLPR vs. one of nine in the low CRF group [χ^2^ = 3.09, *df* = 1, *p* = 0.08]. The four VFD “s” subjects in the “high” CRF group exhibited significantly greater CSF CRF concentrations (pg/ml) than the remaining thirteen subjects [mean (*SD*) = 184.42 (79.80) vs. 93.58 (17.93); *t* = −4.06; *df* = 15, *p* = 0.001, partial eta-squared = 0.51]. These same VFD “s” allele with elevated CSF CRF concentrations subjects exhibited greater amygdala volume vs. the remainder of subjects [mean (*SE*) = 0.40 (0.013) vs. mean (*SE*) = 0.34 (0.007); *F*_(1, 14)_ = 19.94; *p* = 0.0005]. Effects were noted for left amygdala [*F*_(1, 14)_ = 30.75; *p* = 0.00007] and right amygdala [*F*_(1, 14)_ = 6.8 w; *p* = 0.02]. Thus, although the “high” CRF group were not disproportionally represented by VFD subjects, it tended to be populated by “s” allele subjects exposed to VFD, who exhibited both particularly high CSF CRF concentrations and enlarged right and left amygdala volumes.

#### White matter fractional anisotropy

Nine non-VFD and 11 VFD subjects were available for this analysis although we continued to exclude one anomalous non-VFD outlier. Mean ALIC FA inversely associated with amygdala volume [*F*_(1, 16)_ = 7.01; *p* = 0.018]. The relationship between mean anterior limb FA was significant for the left [*F*_(1, 16)_ = 4.87; *p* = 0.04] and right [*F*_(1, 16)_ = 5.67; *p* = 0.03] amygdala.

*Post-hoc* Pearson's correlations revealed significant inverse correlations between mean ALIC FA and left (*r* = −0.49; *N* = 19, *p* = 0.03) and right (*r* = −0.48, *N* = 19, *p* = 0.037) amygdala. Supporting the contention that ELS VFD effects were maintained in this analysis, VFD exhibited reduced mean anterior limb FA compared to non-VFD subjects [VFD mean (*SE*) = 104.38 (9.40) (*n* = 11) vs. non-VFD mean (*SE*) = 139.58 (11.03); *F*_(1, 17)_ = 5.90, *p* = 0.027].

#### Dentate gyrus neurogenesis counts

Doublecortin-immunoreactive cells were counted in the left dentate gyrus of four non-VFD and nine VFD subjects (excluding the one outlier). When combining groups, left hippocampal neurogenesis was negatively related to right amygdala volume [*F*_(1, 10)_ = 5.33; *p* = 0.044] covarying for brain volume. *Post-hoc* Spearman's correlations revealed an inverse correlation for right amygdala (*r* = −0.65; *N* = 13; *p* = 0.015) but not left amygdala (*r* = −0.38; *N* = 13; *p* = 0.198). There were no VFD-rearing effects or 5-HTTLPR × ELS effects for neurogenesis.

#### Associations among divergent measures

Friedman ANOVA and Kendall Coefficient of Concordance for multiple dependent variables were examined to determine the non-parametric association among the various measures affected by ELS. Entering all dependent variables except neurogenesis, the ANOVA Chi Square (*N* = 15, *df* = 3) = 41.00 (*p* < 0.00001) with a coefficient of concordance = 0.91, and average rank *r* = 0.90. When including neurogenesis rates, significance rates are highly similar and remain highly significant. The ANOVA Chi Square (*N* = 11, *df* = 4) = 37.05 (*p* < 0.00001) with a Coefficient of Concordance = 0.84, and average rank *r* = 0.82.

## Discussion

This is the first report demonstrating increased left amygdala size in young adult bonnet macaque males exposed to ELS in the form of VFD rearing. These results are consistent with studies of children reared in institutions (Tottenham and Sheridan, 2009; Tottenham et al., 2010; Malter Cohen et al., 2013), maltreated youth (Whittle et al., 2013), and offspring of chronically depressed mothers (Lupien et al., 2011), suggesting a shared neurodevelopmental mechanisms by which ELS modifies fear response and emotional processing in humans and nonhuman primates. In contrast to left amygdala hypertrophy following ELS in the current report, these same MRI scans reveal both *reduced* left hippocampal volume and *reduced* corpus callosum cross-sectional area in VFD vs. non-VFD subjects (Jackowski et al., 2011). Taken together, the imaging data support the view that amygdala volume is not merely a non-specific effect, but occurs in the opposite direction to other structures which are atrophied by ELS. Although the groups were matched, amygdala volume was not predicted by age or weight of the subjects. Not all studies, particularly in humans, report increased amygdala volume following childhood adversity (Bremner et al., 1999). In certain studies of early abuse, amygdala volume has been found to be decreased (Schmahl et al., 2003). Reasons for these discrepancies are unclear, but include retrospective reporting in humans and timing of stress—the earlier the stress exposure, the more vulnerable the amygdala—and the discrete nature of ELS in animals models compared to humans (Tottenham and Sheridan, 2009). Although there is a suggestion of laterality effects, favoring left amygdala, it should be noted that there were no interactive effects with hemisphere suggesting that significant asymmetry was not present.

Preliminary findings from this study also suggest that the effects of ELS on amygdala volume may be moderated by 5-HTTLPR genotype. Thus, VFD subjects possessing the short allele of the serotonin transporter gene exhibit enlarged amygdala volume in comparison to the other control groups. These findings extend our previous work showing gene by environment (short allele by ELS) effects in nonhuman primates exposed to VFD-rearing for reduced corpus callosum cross sectional area (Jackowski et al., 2011) and CSF CRF concentration elevations (Coplan et al., 2011). Additionally, these reports are consistent with rhesus macaque work from other groups showing a similar short allele by ELS interactions (i.e., peer rearing) on HPA reactivity in response to stress (Barr et al., 2003, 2004; Spinelli et al., 2012). We propose that studies in nonhuman primates provide an essential tool to understand the molecular mechanisms by which ELS interacts with the short allele to modify vulnerability to depression and anxiety in humans.

The amygdala volume measures obtained in this study were also associated with numerous stress-related behavioral and neurobiological parameters previously shown to be altered following ELS. Certain of the groupings we have created do not cleanly divide into VFD vs. non-VFD reared subjects. But in each instance, except neurogenesis, the “stress” state is associated with an increase in right and/or left amygdala volume and an increase frequency (trend for CSF CRF concentrations) of VFD-reared subjects possessing the short allele of the serotonin transporter gene. The focus for the secondary measures report is shifted from a pure ELS effect to the correlates of a range of values in the “stressed” range and their relationship to amygdala volume. We may therefore shed light on the relationship of amygdala volume to stress-related markers. Specifically, enlarged amygdala volume was associated with timid anxiety-like behavior in the masked intruder paradigm, consistent with prior studies showing increased amygdala volume in behaviorally inhibited youth (Hill et al., 2010), and youth exhibiting high scores on the temperamental trait, harm avoidance (Iidaka et al., 2006). Increased amygdala volume was also associated with increased cerebrospinal fluid CRF concentrations, reduced FA of the ALIC, and reduced hippocampal neurogenesis. Because the amygdala provides excitatory glutamatergic projections to the dentate gyrus of hippocampus (Ikegaya et al., 1995, 1996), it is conceivable that increased amygdala volume may be associated with increased excitatory output and excitotoxicity, leading to decreased adult hippocampal neurogenesis (Ikegaya et al., 1995; Abe et al., 1998). However, left hippocampal neurogenesis relates inversely to right but not *left* amygdala volume. This contralateral effect would imply communication across the hippocampal commissure. A reduction in the ventral hippocampal commissure has taken place in human and nonhuman primate phylogeny leading to its near disappearance, whereas the dorsal hippocampal commissure is well developed and represents a significant tract (Gloor et al., 1993). It should be noted that neurogenesis rates do not show a rearing effect or serotonin transporter gene × ELS effect, thus detracting from the view that the neurogenesis effects are related to ELS. We have found that glucagon-like peptide 1, which is elevated following ELS (Kaufman et al., 2007), is strongly related to increased neurogenesis (Coplan et al., 2014), and thus confounds rearing effects on neurogenesis.

The Kendal Coefficient of Concordance, including all study variables, was highly statistically significant, supporting the contention of wide-ranging effects following ELS. Early adversity is not only associated with increased risk for major psychiatric disorders, but for increased risk for a multitude of general health problems as well, with emerging findings suggesting epigenetic mechanisms may be involved in conferring this risk (Yang et al., 2013). The causal pathways leading to these diverse outcomes are currently unknown, and may require embracing complex network-driven, dynamic pathway models, in which context-specific causal relationships can be tested (Schadt et al., 2005).

Limitations of the study include the relatively small number of subjects, particularly for the gene by environment component. However, we acknowledge the preliminary nature of these findings in the hope that the interaction of ELS and the serotonin transporter gene in determining amygdala volume could be explored in larger pre-clinical and clinical samples. Examination of an all male cohort further reduces the generalizability of the research findings and examination of females is warranted and pending. Another limitation was the exclusion of non-VFD left amygdala outlier. However, volume of the amygdala in that subject was >3.4 standard deviations from the group mean and its inclusion would have increased the possibility of a type II error. The ALIC is involved in thalamocortical connections (Coplan et al., 2010) and is not as directly involved with amygdala white matter connections as the uncinate fasciculus, which has previously been implicated in early life adversity (Choi et al., 2012). However, the ALIC is that area which is stimulated during deep brain stimulation (Riva-Posse et al., 2014) suggesting an important role in affective regulation. The study did not correct for multiple comparisons and as such should be regarded as exploratory and requiring replication. Another limitation of the study is the possibility that although an association is demonstrated between ELS and serotonin transporter polymorphism, direct causation or moderation has not and the findings may be due to a secondary as yet undetermined mechanism.

Nonetheless, the current study adds to a growing body of data that suggests ELS is associated with a concert of biological changes that are evident across the lifecycle. The VFD paradigm provides an important animal model to elucidate the cellular and molecular mechanisms by which ELS causes similar changes in humans. For instance, humans, the normal feedback infants receive from their mothers in face-to-face interaction was distorted by having the mothers face their infants but remain facially unresponsive. The infants studied reacted with intense wariness and eventual withdrawal (Tronick et al., 1979). In terms of human extrapolates, Tronick and colleagues (Tronick and Beeghly, 1999) have focused on the effects of poverty severely constricting moment by moment maternal-infant interactions seen as critical to early development, a process we have referred to as maternal “contingent responsivity” (Coplan et al., 1995). Although the biological consequences associated with ELS are frequently long-lasting, they need not be permanent (Weder and Kaufman, 2011). Ongoing multidisciplinary translational research will help to delineate the mechanisms by which ELS confers risk, and help to identify factors to promote resiliency.

### Conflict of interest statement

The authors declare that the research was conducted in the absence of any commercial or financial relationships that could be construed as a potential conflict of interest.
